# Partial Selfing Can Reduce Genetic Loads While Maintaining Diversity During Experimental Evolution

**DOI:** 10.1534/g3.119.400239

**Published:** 2019-07-16

**Authors:** Ivo M. Chelo, Bruno Afonso, Sara Carvalho, Ioannis Theologidis, Christine Goy, Ania Pino-Querido, Stephen R. Proulx, Henrique Teotónio

**Affiliations:** * Instituto Gulbenkian de Ciência, Apartado 14, P-2781-901 Oeiras, Portugal; † cE3c – Center for Ecology, Evolution and Environmental Changes, Faculdade de Ciências, Universidade de Lisboa, Lisboa, Portugal; ‡ Institut de Biologie de l’École Normale Supérieure (IBENS), Inserm U1024, CNRS UMR 8197, F-75005 Paris, France; § Institute of Molecular Biology and Biotechnology, Foundation for Research and Technology-Hellas, 73100 Heraklion, Greece; ** Leibniz Research Institute for Environmental Medicine, 40225 Düsseldorf, Germany, and; †† Department of Ecology, Evolution, and Marine Biology, University of California Santa Barbara, CA 93106

**Keywords:** Self-fertilization, Disequilibrium, Evolve & Resequence, Overdominance, *C. elegans*

## Abstract

Partial selfing, whereby self- and cross- fertilization occur in populations at intermediate frequencies, is generally thought to be evolutionarily unstable. Yet, it is found in natural populations. This could be explained if populations with partial selfing are able to reduce genetic loads and the possibility for inbreeding depression while keeping genetic diversity that may be important for future adaptation. To address this hypothesis, we compare the experimental evolution of *Caenorhabditis elegans* populations under partial selfing, exclusive selfing or predominant outcrossing, while they adapt to osmotically challenging conditions. We find that the ancestral genetic load, as measured by the risk of extinction upon inbreeding by selfing, is maintained as long as outcrossing is the main reproductive mode, but becomes reduced otherwise. Analysis of genome-wide single-nucleotide polymorphisms (SNPs) during experimental evolution and among the inbred lines that survived enforced inbreeding indicates that populations with predominant outcrossing or partial selfing maintained more genetic diversity than expected with neutrality or purifying selection. We discuss the conditions under which this could be explained by the presence of recessive deleterious alleles and/or overdominant loci. Taken together, our observations suggest that populations evolving under partial selfing can gain some of the benefits of eliminating unlinked deleterious recessive alleles and also the benefits of maintaining genetic diversity at partially dominant or overdominant loci that become associated due to variance of inbreeding levels.

Classic theoretical models posit a key role for deleterious recessive alleles in the evolution of reproductive mode modifiers (Lande and Schemske 1985; Charlesworth and Charlesworth 1987; Charlesworth *et al.* 1990). Under self-fertilization (selfing), because more homozygotes are produced than under cross-fertilization (outcrossing), there are larger fitness differences between individuals and thus more opportunity for purifying selection. If mutation rates are not high enough to result in extinction (Lande *et al.* 1994; Schultz and Lynch 1997), then predominant selfing should evolve. In this case, populations will be mostly free of deleterious recessives but will have reduced standing diversity, which may compromise future adaptation. Otherwise, mutational load can become very high and predominant outcrossing should evolve with populations maintaining deleterious recessives that could become beneficial under environmental change.

Although classic models suggest the evolution of predominant selfing or predominant outcrossing should be common, comparative studies in plants and in animals have found that a number of natural populations show mixed selfing and outcrossing at intermediate frequencies (Goodwillie *et al.* 2005; Jarne and Auld 2006). To explain the maintenance of such partial selfing, several models have been developed to deal with the particular way by which partial selfing structures genetic diversity between individuals and determines the maintenance of genetic loads – here defined as the fitness difference between the focal population and genotypes free of deleterious variation. One of the consequences of selfing is that linkage disequilibrium is increased and thus effective recombination reduced (Nordborg *et al.* 1996). Partial selfing further creates identity disequilibrium, as individuals vary in their inbreeding coefficients because their immediate ancestors have experienced a variable number of generations under selfing or outcrossing (Haldane 1949; Weir and Cockerham 1973). With identity disequilibrium, heterozygotes tend to be associated more often with other heterozygotes, and homozygotes with other homozygotes, than that expected with random mating or exclusive selfing. When highly deleterious alleles are completely recessive, linkage and identity disequilibrium can prevent the selective purging of genetic loads as outcrossed progeny are much more viable than selfed progeny (Lande *et al.* 1994; Bierne *et al.* 2000; Kelly 2007; Roze 2015). There is usually a sharp threshold in selfing rates below which populations behave as if they were outcrossing and maintain genetic loads, and above which deleterious recessives are selectively purged (Lande *et al.* 1994; Roze 2015), although partial selfing is not found at mutation-selection balance. This leaves more complex scenarios, such as selection on deleterious alleles of variable dominance together with selection on overdominant loci in the presence of significant linkage and/or identity disequilibrium to explain partial selfing, although such scenarios are in need of further investigation, *cf*. Ziehe and Roberds (1989); Charlesworth *et al.* (1990); Johnston *et al.* (2009); Roze (2015, 2016).

During the last 10 years the nematode *Caenorhabditis elegans* has become a good experimental model to study how standing genetic variation for fitness depends on reproductive mode (Teotónio *et al.* 2017). *C. elegans* is an androdioecious species, where hermaphrodites are capable of autonomous selfing and of outcrossing with males, but incapable of outcrossing with other hermaphrodites. Sex determination is chromosomal with hermaphrodites having two sex chromosomes and males only one (XX and X0). For these reasons, and because sex segregates in a Mendelian fashion (Teotónio *et al.* 2012), one minus twice the frequency of males is a good proxy for the selfing rate of a population (Stewart and Phillips 2002). Wild isolates of *C. elegans* are usually devoid of males (Teotónio *et al.* 2006), are highly inbred and show outbreeding depression when crossed with each other (Dolgin *et al.* 2007; Chelo *et al.* 2013). Outbreeding depression is due to the fact that a long history of predominant selfing, metapopulation dynamics and linked selection likely led to the evolution of gene complexes that decrease fitness when disrupted (Cutter 2006; Seidel *et al.* 2008; Rockman *et al.* 2010; Andersen *et al.* 2012; Gaertner *et al.* 2012).

While in nature selfing is the predominant mode of reproduction, in the laboratory populations can maintain partial selfing. When experimental evolution starts from populations resulting from the hybridization of wild isolates, partial selfing is stably maintained for up to 100 generations (Anderson *et al.* 2010; Teotónio *et al.* 2012; Masri *et al.* 2013; Carvalho *et al.* 2014a). Our previous work suggests that partial selfing is maintained because purging of partially recessive deleterious alleles, which had been generated through the disruption of natural gene complexes, co-occurs with selection of overdominant loci, leading first to a reduction in genetic loads and then to the maintenance of excess heterozygosity (Chelo *et al.* 2013; Chelo and Teotónio 2013). However, our conclusions had to be tempered because of problems in experimental design, as we assumed that biparental and uniparental inbreeding were equivalent, and we lacked a control differentiating reduced effective recombination because of increased linkage or identity disequilibrium.

Here, we analyze the *C. elegans* evolution experiments first reported in Theologidis *et al.* (2014), where populations with different reproduction systems, obtained by introgression of sex determination mutant alleles into a lab domesticated population (Teotónio *et al.* 2012), were cultured for 50 generations in challenging NaCl conditions (Figure 1). We ask how androdioecious populations with partial selfing cope with extreme inbreeding and the potential for extinction at very small population sizes when compared with monoecious and trioecious populations. Monoecious populations only have hermaphrodites and thus reproduction is exclusively done by selfing, whereas trioecious populations have males, females and a small number of hermaphrodites and thus reproduction is predominantly done by outcrossing.

**Figure 1:**
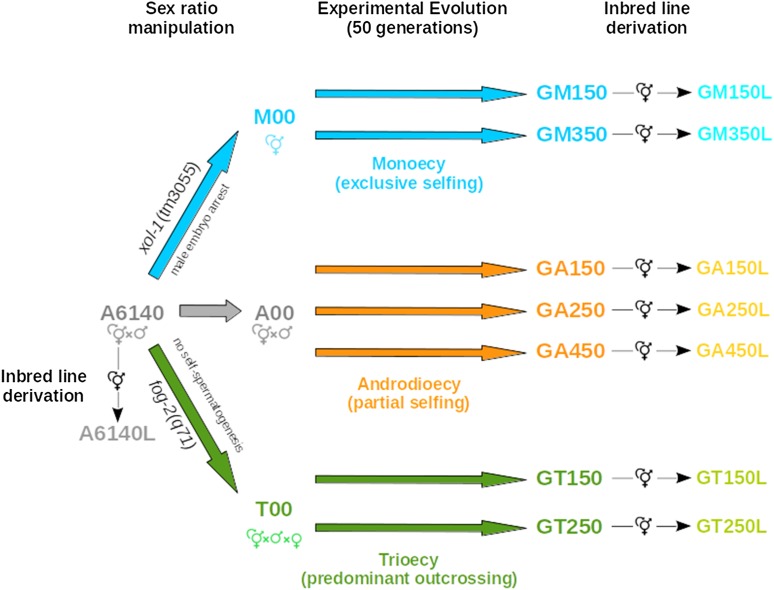
Experimental design and origin of populations used in this work. The genetically diverse androdioecious population A6140 (containing males and hermaphrodites) was used as the ancestral population for experimental evolution under increasing NaCl concentrations for 50 generations, reported as the ”Gradual regime” in Theologidis *et al.* (2014). This ancestral population was subjected to introgression of sex determination mutant alleles to generate populations with different proportions of males, hermaphrodites and females, which evolved under different contributions of selfing and outcrossing (M00 - monoecious; A00 - androdioecious; and, T00 - trioecious populations). Population A00 was derived by sampling of A6140 without any genetic manipulation. Experimental evolution was done with several replicates and, at the end of it, inbred lines were derived by selfing from the resulting populations as had been for the ancestral A6140. Population nomenclature follows Theologidis *et al.* (2014) and Noble *et al.* (2017) and font colors are those used throughout this paper.

To measure genetic loads, we follow the extinction rates of lineages derived from evolving populations by extreme inbreeding (selfing), and also the fertility of surviving inbred lines (Figure 1). The risk of extinction and fertility of monoecious populations is the best estimate of the fitness of “optimal” genotypes, given that exclusive selfing during evolution quickly reduces heterozygosity (Guzella *et al.* 2018). On the other hand, the risk of extinction and fertility of trioecious populations benchmark the genetic load of the ancestral population, since at the small effective population sizes ($10^{3}$) and time frame employed mutation is not expected to contribute much to the observed responses (Matuszewski *et al.* 2015). Genome-wide single nucleotide polymorphism (SNP) diversity of the lines surviving inbreeding is further compared with that of the evolving populations. We do this to find how selection on deleterious variation depends on linkage disequilibrium or on the identity disequilibrium generated by partial selfing.

## Materials and Methods

### Ancestral population

All populations in this study are ultimately derived from a hybrid population obtained by a funnel-crossing scheme where 16 parental wild isolates were reciprocally crossed two-by-two, and their hybrid offspring subsequently crossed also in a pairwise fashion, until obtaining the final hybrid population (Teotónio *et al.* 2012). The hybrid population was then cultured under 4-day discrete and non-overlapping life-cycles, following the “bleach/hatch-off” protocol of Stiernagle (1999), by using a sodium hypochlorite (bleach) solution to kill all adults and larvae and let only unhatched embryos survive. Census population sizes of $10^{4}$ between the first larval stage and the time of reproduction were maintained to derive a 140 generation lab-domesticated population (Teotónio *et al.* 2012; Theologidis *et al.* 2014) and at the effective population sizes of $10^{3}$ (Chelo and Teotónio 2013). During lab domestication, male frequencies were stable at about 25%, thus indicating that selfing was maintained at 50% given that hermaphrodites cannot mate with each other (Teotónio *et al.* 2012). The 140-generation lab-adapted population (named A6140) is the androdioecious ancestor population for all the experimental populations reported (see Figure 1).

### Genetic manipulation of sex ratios

The genetic manipulation of sex ratios in A6140 has been detailed in Theologidis *et al.* (2014). We mass introgressed an X-chromosome sex determination mutant allele [*xol-1**(**tm3055**)*] into A6140, a mutant impairing X-chromosome dosage compensation and leading to the arrest of male development, to derive a monoecious population composed exclusively of hermaphrodites [homozygous for *xol-1**(**tm3055**)*] that only reproduce by selfing (named M00). In parallel, we mass introgressed an autosomal sex determination recessive mutant allele [*fog-2**(**q71**)*] into A6140, a mutant that eliminates self-spermatogenesis in hermaphrodites, to derive a trioecious population composed of males and females [homozygous for *fog-2**(**q71**)*], and hermaphrodites [homozygous for *fog-2**(wt)*] at an initial frequency of $10^{-2}$ (named T00). This population predominantly outcrossed during experimental evolution, despite the effects of high salt in delaying development (Theologidis *et al.* 2014). To generate an androdioecious population (named A00), with a comparable initial genetic diversity to M00 and T00, we sampled the same number of families from A6140, as those during the derivation of M00 and T00. As measured with competition assays against a tester strain (Teotónio *et al.* 2017), initial population-fitness differences between M00, T00 and A00 are explained by differences in sex ratios, as all individuals in the M00 population are hermaphrodites whereas this number is reduced to half in the A00 population and to negligible numbers in the T00 population (in Theologidis *et al.* (2014) see Figure S5 for fitness data in ancestral populations).

### Experimental evolution

The evolution experiment has also been previously reported in Theologidis *et al.* (2014) and the populations here described were those evolving under the conditions defined for the “Gradual regime”. Replicate populations of M00, T00 and A00 were exposed in NGM-lite media to 8 mM/generation increases in NaCl concentration, from the first larval stage until adulthood during 35 generations, after which they were kept at 305 mM NaCl for 15 additional generations. Here, we report results of 2 replicates derived from T00 (GT150, GT250; nomenclature according to Theologidis *et al.* (2014)), 2 replicates from M00 (GM150, GM350), and 3 replicates from A00 (GA150, GA250, GA450), all after 50 generations of experimental evolution (Figure 1). In Theologidis *et al.* (2014) a total of four replicates were used for experimental evolution but, for some, inbred line derivation was not performed or fertility and genotypic data were not obtained. Fitness measurements obtained with competition experiments show that adaptation to the high salt environment was similar under the three reproduction systems by generation 35, and that possible differential sexual selection was not detected during this period [Figure 4D-F in Theologidis *et al.* (2014)].

### Genetic load: inbred line derivation and extinction assays

For line derivation, we followed standard protocols similar to mutation accumulation evolution experiments, where population sizes are kept to a minimum to allow fixation of segregating alleles in the population within each line in mostly a neutral fashion (Teotónio *et al.* 2017). In our case, population sizes were of N=1, because we derived lines by selfing of hermaphrodites. In this situation, we expect that alleles with an expected average selection coefficient of s<1/2N=0.5 will be randomly fixed within each line within 3-4 generations, assuming log additive effects and no initial linkage between them (Kimura 1983). Given the small population sizes, and the duration of the assays, very few if any new mutations should appear and get fixed during line derivation (Vassilieva *et al.* 2000; Estes *et al.* 2004).

In our experiments, we used trioecious populations, instead of exclusively outcrossing (dioecious) populations so that we could derive inbred lines by selfing from all experimental populations and thus not confound potential selection under biparental and uniparental inbreeding as in Chelo *et al.* (2013). Specifically, A6140, and all generation 50 populations, were revived from frozen stocks, each with >10^3^ individuals, and cultured for two generations in a common environment. At the third generation, hermaphrodites were handpicked at the larval immature L3-L4 stage and placed in individual wells of 12-well cell culture plates, previously filled with NGM-lite media and *E. coli* bacteria as *ad libitum* food. During inbred line derivation we used 25 mM NaCl, the salt condition of prior lab domestication during 140 generations. Every 4 to 7 days, one L3-L4 offspring hermaphrodite was handpicked and placed into a fresh well, consecutively for 16 generations for A6140 or for 13 generations in all other populations. Resulting lines were expanded in numbers for 2-3 further generations and cryogenically frozen. During inbred line derivation, the 12-well plates were kept at $4^{o}C$ to prevent growth until successful offspring production could be confirmed in the following generation. A line was considered extinct if reproduction from the previous generation failed twice. Because of this recourse to backups the effective population size during line derivation was slightly above N=1. This procedure, of line derivation, was carried out several times (in “blocks”) always starting from cryogenically frozen experimental populations’ stocks until the final number of lines was obtained. For A6140 this was done in two blocks and for generation 50 populations in another six separate blocks, where in each block lines were derived in parallel from each of the evolved populations.

### Genetic load: fertility assays

Data from the inbred lines in low salt (25 mM NaCl), the environment where previous lab domestication and line derivation was done, was previously reported in Noble *et al.* (2017). Here, we present new data from the inbred lines in the target high salt environment (305 mM NaCl) and from all populations before line derivation in both high and low salt. Briefly, hermaphrodites were individually placed at the L3-L4 larval stage into wells of a 96-well assay plate with NGM-lite media and bacteria, and 24h later exposed to the bleach solution. Hatched live L1 larvae were counted 24h after bleach exposure. This assay closely mimics the culture protocol used during experimental evolution with the exception of density and opportunity for mating, which, during experimental evolution are those of 1000 individuals growing in 90 mm (diameter) Petri dishes. Individual fertility was measured in all experimental populations (A6140, A00, M00, T00, and all generation 50 derived populations, see Figure 1) and the inbred lines at 25 mM and 305 mM NaCl (total n = 40800). Manual curation was done during the assay and during image analysis by removing data where individual hermaphrodites could not be found (n = 917) or were dead (n = 1273) at the time bleach exposure (because worms desiccated on the walls of the plate wells, for example). Cases were males had been inadvertently picked instead of hermaphrodites (n = 449) or more than 1 individual was found were also removed (n = 354), as well as whenever bacteria was seen to have not been seeded (n = 807), to prevent results from being affected by lack of food during final stages of development. Data from wells where the adult hermaphrodite was still alive after bleach/hatch-off were also discarded to prevent an upward bias on estimates due to a protocol’s inefficient implementation.

### DNA collection and genotyping

Genomic DNA from A6140 and each of the G50 populations was extracted from individuals with the ZyGem prepGEM Insect kit and from pools of individuals from each inbred line using the Qiagen Blood and Tissue kit. A total of 830 SNPs distributed evenly across genetic distance, which was estimated by linear interpolation of the expected F2 map distances from Rockman and Kruglyak (2009), were genotyped using Sequenom methods (Bradic *et al.* 2011). The choice of the SNPs was based on known variation between the founder wild isolates used to create the A6140 population as described in (Noble *et al.* 2017). For the ancestral and evolved populations, each individual was genotyped for chromosomes I and II, III and IV, and V and X, respectively. Quality control was done following the criteria of Chelo and Teotónio (2013), with the resulting genotype matrix being composed of 743 SNPs. Sample sizes after quality control can be found in Supplementary Table 1.

All genotype data for the lines has been previously published in Noble *et al.* (2017) to cross-validate the whole-genome line sequencing done in that study. Data for the monoecious populations at generation 50 has been published in Guzella *et al.* (2018). New genotype data are presented for the ancestral population (A6140) and the trioecious and androdioecious populations at generation 50.

### Genetic diversity during experimental evolution

In ancestral and evolved experimental populations, SNP allele diversity was estimated as $H_{e}=2pq=1-\left(p^{2}+q^{2}\right)$, and an inbreeding coefficient (fixation index) as $F_{is}=1-\left(H_{o}/H_{e}\right)$, with $H_{o}$ being the observed proportion of heterozygotes (Crow and Kimura 1970). To avoid sampling biases, SNPs with $H_{e}$ lower than 0.05 were removed prior to calculation of $F_{is}$.

Linkage disequilibrium between pairs of SNPs was estimated as $r^{2}=D^{2}/p_{i}\left(1-p_{i}\right)p_{j}\left(1-p_{j}\right)$, where D is the difference in genotypic frequencies from that expected given the allele frequencies $p_{i}{,}_{j}$ in SNPs i and j (Weir 1996). Comparisons between reproductive systems and the ancestral population were done with replicate as a random factor, via nonlinear regression using *nlme* R package (Pinheiro *et al.* 2018), with the following input formula for the *nlme* function:

R2∼I(1/(1+4∗x∗c)), fixed=x∼PopType, random=x∼1|PopReplicate

With this, we model the observed $r^{2}$ (“R2” in code) for every pair of SNPs as a function of *c*, which is the genetic distance between SNPs, and *x*, which determines the rate of LD decay and can differ between populations (“PopType” - ancestral, trioecious, androdioecious and monoecious). The underlying assumption is that the expected decay of linkage disequilibrium with genetic distance follows the equation given by Sved (1971) as $E\left(r^{2}\right)=1/\left(1+4Nc\right)$, where *N* refers to the effective population size. Only SNPs segregating at frequencies between 0.05 and 0.95 were used.

Identity disequilibrium, g2 (David *et al.* 2007), was calculated using the R package *inbreedR* (Stoffel *et al.* 2016). Identity disequilibrium is only estimated for populations where partial selfing is possible, that is, the androdioecious populations and trioecious populations.

Finally, we also calculated the mean effective number of haplotypes ($h_{e}$) as a measure of multi-allelic diversity: $1/\sum k_{i}^{2}$, with $k_{i}$ being the proportion of haplotype *i* in a given replicate population or group of lines. $h_{e}$ can be interpreted as the number of haplotypes segregating in the population if they were all present at the same frequency (Crow and Kimura 1970). For each population, haplotypes are defined in non-overlapping windows of 10 SNPs, as diploid phased genotypes (Chelo and Teotónio 2013), independently for each population. Varying SNPs per window between 5 and 20 does not affect the conclusions (not shown).

For all diversity metrics, we only show the genome wide average in each population.

### Genetic diversity among inbred lines

$H_{e}$ and $h_{e}$ metrics were calculated for the surviving inbred lines and used to evaluate how diversity changed after the 13-16 generations of selfing. For that purpose, means and standard deviations based on single individuals from the experimental populations were compared with the means and standard deviations of single lines (obtained from pools of individuals). Each line was considered as a diploid individual. Due to the sampling design, genetic data from 16 individuals was available from the experimental populations, whereas the final number of lines was usually above 50. To prevent biases arising from unequal sample sizes, we calculated the metrics in the inbred lines as the average of subsets with 16 observations, as obtained by Jackknife resampling.

Following Chelo *et al.* (2013), to provide neutral confidence intervals for the expected genetic diversity metrics after inbreeding, numerical simulations were run with selfing for 13 (for generation 50 populations) or 16 generations (for A6140). Less diversity after inbreeding, among surviving lines, in comparison with the corresponding populations, indicates purifying selection of highly deleterious alleles during the assay. More diversity indicates that either overdominant loci or deleterious alleles from different loci in repulsion linkage disequilibrium (associative overdominance, (Ohta and Kimura 1970; Palsson and Pamilo 1999)), are present in the populations from where the lines are derived. Conditional on the observed diversity before line derivation, 1000 simulations were run per population. All chromosomes were separately analyzed and metrics among SNPs averaged across chromosomes. Each run started by randomly sampling phased diploids, with replacement. Meiotic crossover was simulated by exchanging consecutive sets of alleles between the two parental haplotypes, and assuming complete crossover interference and F2 map sizes of 50cM per chromosome. Crossover occurred randomly between two consecutive SNPs depending on the probability given by the genetic distances between them as in Rockman and Kruglyak (2009), and zygotes obtained by joining two independent gametes from the same individual. Similar sampling of lines was done from the simulations as that in the assays, where usually more than 50 lines survived inbreeding.

### Statistical analysis

Testing for differences in the several genetic diversity metrics was done with ANOVA using reproductive system as the predictor variable. Because we have only one data point for the ancestral population, we fixed the intercept in these models. Two-sided Tukey *t*-tests were then used for post-hoc comparisons using the *lsmeans* package in R (Lenth 2015). The exception was in the analysis of identity disequilibrium, where individual *t*-tests were uncorrected. P-values from significant tests are reported as: * p<0.05 or ** p<0.005. Shapiro-Wilk and Bartlett’s tests were done to check for departure from normality and homocedasticity of residuals, respectively. Maximum likelihood parameter estimates obtained by nonlinear regression of $r^{2}$ decay with genetic distance were compared against the ancestral population via a Wald test.

The risk of line extinction per generation was assessed with Kaplan-Meier estimators (Kaplan and Meier 1958), assuming right censored data. Cox proportional hazards regression models (Cox 1972) were then used to compare the extinction risks of each population type (ancestral, evolved trioecious and evolved androdioecious) against the monoecious evolved populations (as it provides the expected line extinction due to environmental effects, assuming individuals were homozygous prior to line derivation), or between evolved populations and the ancestral population. In these models there is a baseline risk (hazard ratio, HR) for each line undergoing selfing, which can differ between lines of different treatments in a time-independent manner. We employed a mixed effects model for analysis, with reproductive system as the fixed factor and replicate population as a random factor. Z-scores were used for significance testing. The R package *coxme* was used for computation (Therneau 2015).

For fertility data, before data analysis, we noticed a high number of infertile hermaphrodites and females. This occurred because most of them were either not mated when individually transferred 24h before reproduction and/or because they had not reached maturity at the time of the assay. For this reason we decided to first model fertility distributions assuming Poisson, negative binomial, or zero-inflated errors for all populations and inbred lines (using R functions *glm* in the stats package, *glm.nb* in the MASS package, and *zeroinfl* in the pscl package). Likelihood ratio tests were employed for model selection. Zero-inflated negative binomial error distributions were chosen for subsequent analyses as they provided the best fit. We then employed generalized mixed effect models separately for 25 mM and 305 mM NaCl conditions, using the function *glmmadmb* from package glmmADMB (Skaug *et al.* 2013). We tested for the effects of experimental evolution (ancestral *vs.* evolved) and the reproductive system at generation 50, with assay blocks and replicate populations as random factors. Differences between experimental populations and the average of inbred lines derived from them was again carried out with the *lsmeans* and *pairs* functions in R (Lenth 2015).

### Data availability

Fertility and genotype data for the inbred lines associated with Noble *et al.* (2017) has been archived in FigShare 10.6084/m9.figshare.5326777. All data and R code for analysis can be found in FigShare accessions: 10.6084/m9.figshare.5539606 (readme), 10.6084/m9.figshare.5539612 (code), 10.6084/m9.figshare.8665661 (dataset phenotypes and SNP genotypes), 10.6084/m9.figshare.5539618 (dataset haplotypes), and 10.6084/m9.figshare.5539627 (dataset simulated haplotypes). Supplemental material available at Figshare: https://doi.org/10.25387/g3.8397128.

## Results

### Selfing rates during experimental evolution

As previously noted (Theologidis *et al.* 2014), delayed development at high salt concentrations (above 250 mM NaCl) resulted in diminished opportunity for mating. This led to outcrossing impairment and, consequently, to androdioecious populations showing a decrease in males from initially > 40% until generation 30 to 10% by generation 50 (Supplementary Figure S1). Despite delayed development, trioecious populations maintained above 40% of males and about 40% of hermaphrodites by generation 50. Under trioecy, hermaphrodites provide reproductive assurance by selfing and they replace females at high salt concentrations. Yet, their starting low numbers and the more favorable conditions during the first 30 generations imply that males are nevertheless maintained at high frequencies, and outcrossing (between males and both females and hermaphrodites) is the predominant reproductive mode. In these populations, by the time stressful salt concentrations start to be experienced (from generation 30 onwards), most hermaphrodites carry the *fog-2**(**q71**)* mutant allele in the heterozygous state, meaning they will also produce females through selfing, and male mating ability has had the opportunity to improve through sexual selection (Theologidis *et al.* 2014). Both factors contributed to the maintenance of outcrossing and males at higher frequencies than in androdioecious populations despite high salt conditions.

### Evolution under predominant outcrossing or partial selfing maintains standing genetic variation

**Figure 2:**
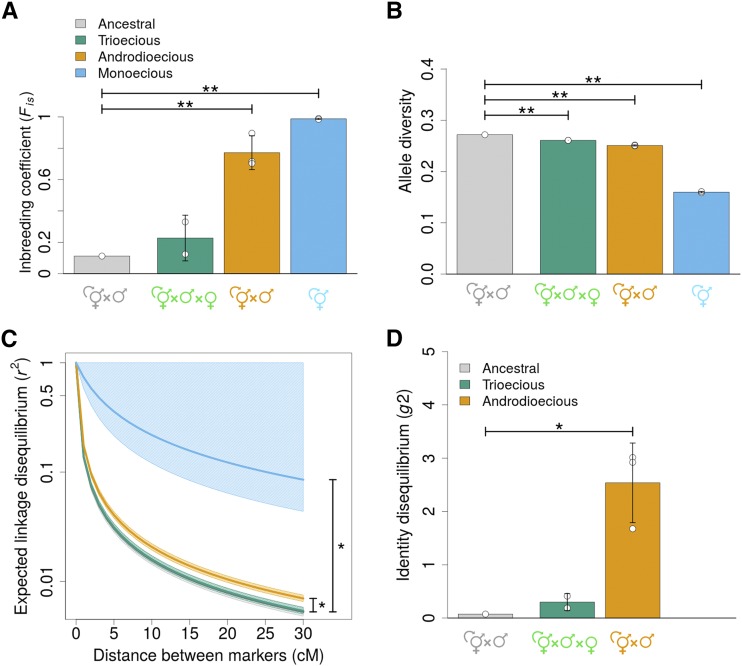
Genetic diversity comparison between the ancestral population and populations evolved under the three different reproductive modes. Legend colors apply equally to all panels. (a, b), Higher inbreeding coefficients ($F_{is}$) are seen in monoecious and androdioecious populations than in the ancestral or trioecious populations, while SNP allele diversity ($H_{e}$) is almost halved only in monoecious populations. (c), Linkage disequilibrium within chromosomes, shown as the expected decay in $r^{2}$ with genetic distance (cM). Data were fitted with $E\left[r^{2}\right]=1/\left(1+xc\right)$, with respect to *x* and lines represent fitted values for ancestral and derived populations with shaded areas showing 95% confidence intervals (see Materials and Methods). Lines from the ancestral and trioecious populations overlap. Y axis is shown in a logarithmic scale. (d), Expected identity disequilibrium (g2) is shown with exception of monoecious populations that have no heterozygotes. Means and one SD among replicate populations (dots) are shown (see Materials and Methods). Asterisks show significant differences between experimentally evolved populations and the ancestral population for p-values < 0.05 (*) or p-values < 0.005 (**).

When compared with the ancestral population, androdioecious and monoecious populations reveal an increase in inbreeding coefficients ($F_{is}$; from 0.11 to 0.77 +/− 0.110 SD; Tukey $t_{4}$ = 5.42, p-value = 0.01, for the androdioecious populations; and from 0.11 to 0.988 +/− 0.003 SD for the monoecious populations; $t_{4}$ = 6.77, p-value = 0.006), unlike the trioecious populations, which maintain ancestral levels (Figure 2a). In monoecious populations, this increase in inbreeding coefficients was accompanied by a marked reduction in SNP allele diversity after 50 generations of experimental evolution (Figure 2b; $t_{4}$ = -91.45, *P* < 0.0001), a result that contrasts with values from androdioecious populations which, together with trioecious populations, only show a small decrease in allele diversity (Figure 2b; for trioecious *vs.* ancestral $H_{e}$ comparisons: $t_{4}$ = -8.98, p-value = 0.002; for androdioecious *vs.* ancestral: $t_{4}$ = -18.19, p-value = 0.0001).

### Evolution under partial selfing maintains ancestral linkage disequilibrium but increases identity disequilibrium

Linkage disequilibrium decays with genetic distance in a similar fashion in the ancestral and trioecious populations ($r^{2}$; Figure 2c). In contrast, androdioecious and especially monoecious populations show increased linkage disequilibrium ($W_{z}$ = - 4.4, df = 2, p-value = 0.047, for the androdioecious populations; and $W_{z}$ = -16.3, df = 1, p-value = 0.03, for the monoecious populations). Identity disequilibrium increased in androdioecious populations relative to the ancestral population (Figure 2d; g2 increased from 0.07 to 2.5 +/− 0.8 SD; Student $t_{2}$ = 5.71, p-value = 0.029). Trioecious populations maintain the same identity disequilibrium as that observed in the ancestral population (Student $t_{1}$ = 1.15, p-value = 0.454). Identity disequilibrium is undefined in monoecious populations because individuals are homozygous at the measured SNPs ($H_{o}=0.002$).

### Evolution under predominant or partial selfing reduces genetic loads

**Figure 3:**
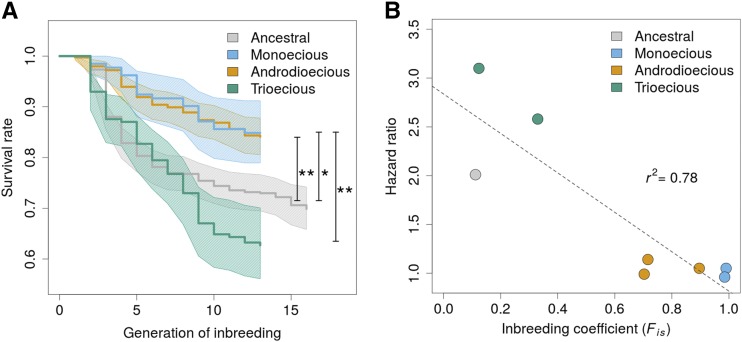
(a), Kaplan-Meier estimates of survival rates (1 minus risk of lineage extinction) are shown for the laboratory adapted ancestral population (gray line) and the experimentally evolved populations, with shaded areas representing 95% confidence intervals. Hermaphrodites were selfed for 13-16 generations and the proportion of surviving lineages recorded. Simulations of the extent to which lineage extinction in the trioecious population is due to picking segregating females or *fog-2(q71)* heterozygous hermaphrodites is shown in Supplementary Figure S2. Significant differences in the risk of extinction to evolved monoecious populations, which represent the condition of no inbreeding depression, or ancestral population are shown as lines: * p-value < 0.05, ** p-value < 0.005. In (b), the tendency for populations with higher inbreeding after 50 generations of experimental evolution to have a lower risk of extinction is revealed by a negative correlation (p-value < 0.005) between inbreeding coefficients and hazard ratios, in which the monoecious populations were used as reference (shown in blue). Coefficient of determination is shown.

Given the absence of heterozygotes in the monoecious populations, the observed 85% lineage survival during inbreeding provides the benchmark for lack of a genetic load and should reveal the risk of extinction due to environmental factors. Survival of monoecious populations upon inbreeding contrasts with the ancestral population where only 70% of the lines survived after 16 generations of inbreeding (monoecious *vs.* ancestral hazard ratio HR = 2.0, p-value = 0.014; Figure 3a). Androdioecious populations likewise evolved a lower risk of extinction upon inbreeding when compared to the ancestral population (HR = 0.53, p-value = 0.002). Both androdioecious and monoecious populations have similar extinction risks upon inbreeding (HR = 1.05, p-value = 0.84), despite the apparent difference in inbreeding coefficients between them (Figure 3b).

Experimental evolution under predominant outcrossing (trioecy) did not result in a reduction of the ancestral genetic load. Trioecious populations maintained the ancestral risk of extinction (Figure 3a; HR = 1.41, p-value = 0.1), while maintaining the same inbreeding coefficients (Figure 2b). When compared with monoecious populations, trioecious population have an increased risk of line extinction (HR = 2.82, p-value = 0.0002). Because trioecious populations segregate a large proportion of females and derivation of inbred lines was done by selfing of single individuals, extinction could simply be due to their inadvertent hand-picking during initial assay set-up. Numerical simulations show, however, that that is not the case (Supplementary Figure S2).

**Figure 4:**
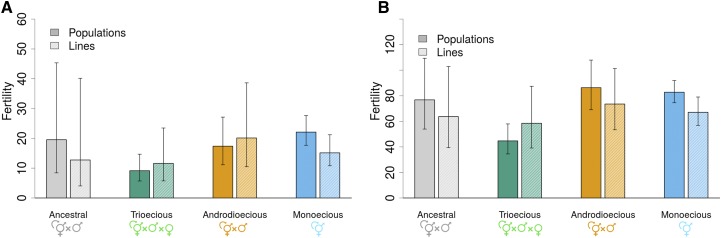
Reproductive output and line derivation. Mean fertility and one SEM among replicate populations are shown for populations from each reproductive mode, in 305 mM NaCl (a) and 25 mM NaCl (b). In each group, from left to right, results are shown for the experimental populations and the inbred lines derived from them. No significant differences between populations and inbred lines were found.

We also attempted to detect genetic loads by comparing fertility between experimental populations and the inbred lines derived from them, given that a difference between the mean values of fitness components in populations and the mean value among the surviving inbred lines would indicate inbreeding (negative difference) or outbreeding (positive difference) depression (Lynch and Walsh 1998). Moreover in the case of a negative difference between the means, if some lines have fertility values above those shown by the population, one can infer that they have “purged” deleterious recessive alleles during inbreeding by selfing, while lack of lines with fertility values above the average fertility of individuals in the population is consistent with both purging of deleterious recessive alleles and overdominant selection during line derivation (Lynch and Walsh 1998, p. 283). During experimental evolution fertility increased, particularly for trioecious and monoecious populations in high salt conditions (Supplementary Figure S3). This is a result that is consistent with adaptation to high salt, as in Theologidis *et al.* (2014). There is, however, little evidence that fertility in the inbred lines is different from the populations they were derived from (Figure 4).

Given that inbred line derivation was done at low salt, the observed line extinction differences between reproduction systems might not be representative of fitness differences during experimental evolution. This problem does not appear to be of much concern because we detected a positive genetic correlation in fertility across the two salt environments for the androdioecious populations and no obvious trade-off between high and low salt in the other reproduction systems (Supplementary Figure S4).

### Predominant and partial outcrossing populations are more diverse after inbreeding than expected

**Figure 5:**
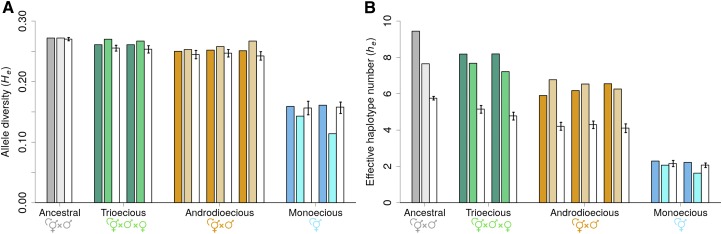
Diversity, shown as the average SNP allele diversity ($H_{e}$, panel a) or mean effective haplotype number ($h_{e}$, panel b) is plotted for each replicate population (A6140, GT150, GT250, GA150, GA250, GA450, GM150 and GM350 - dark-colored bars) and among the inbred lines derived from them (A6140L, GT150L, GT250L, GA150L, GA250L, GA450L, GM150L and GM350L - light-colored bars). Adjacent to the inbred lines (in white), the expected diversity with no selection during enforced inbreeding obtained with 1,000 numerical simulations is shown with error bars giving 95% credible intervals (see Materials and Methods).

SNP and haplotype diversity metrics were calculated among the lines that survived inbreeding by selfing in order to detect selection during inbreeding. In trioecious and androdioecious populations, simulations of genetic drift during inbreeding and sampling predict that SNP diversity among lines is maintained or slightly reduced whereas haplotype diversity is always expected to be reduced (Figure 5). However, observed results show that trioecious and androdioecious populations have higher diversity levels than that expected with the simulations, which are in line with observations of the ancestral population. In contrast, monoecious populations show a larger reduction of SNP and haplotype diversity after inbreeding than expected. For monoecious populations, the simulations predict that diversity is maintained or reduced with inbreeding.

Further evidence for strong selection during the inbreeding assay comes from the SNP frequency changes observed during experimental evolution (Supplementary Figure S5). For trioecious and androdioecious populations, there is a negative correlation in differentiation during experimental evolution and inbreeding, while in monoecious populations only a much weaker relationship could be found.

## Discussion

In this study, we found that genetically diverse populations of *C. elegans* reproducing by selfing for 50 generations evolved reduced risk of extinction when maintained at very small sizes and under extreme inbreeding conditions. This result, which is consistent with the reduction of an ancestral genetic load, was likely caused by selection on viability loci given the similar reproductive outputs that were found in populations and among the surviving lines after inbreeding appear to rule out fertility as a major fitness component. Trioecious populations, which reproduced predominantly by outcrossing, had the same risk of extinction upon extreme inbreeding as the ancestral population, suggesting that they maintained the ancestral genetic load. We also found that populations that experienced some degree of outcrossing during experimental evolution (trioecious and androdioecious populations) maintained more genetic diversity than that expected solely under genetic drift, or under genetic drift and directional selection.

Theory on the evolution of selfing rates provide some indication on the possible genetic underpinnings of fitness variation and how partial selfing might have influenced it. If genetic loads – defined as the potential loss of fitness relative to a genotype free of deleterious genetic diversity – are generated by unlinked and partially to fully recessive deleterious alleles, and with selection acting independently at each locus, then one of two stable outcomes is possible (Charlesworth *et al.* 1990; Lande and Schemske 1985). One possibility is that predominant selfing evolves, with populations concomitantly becoming mostly free of deleterious diversity because homozygous individuals are efficiently selected against. The other possibility is that high levels of outcrossing evolve, with populations bearing a load of deleterious recessive alleles sufficient to avoid extinction, as selection is less efficient in purging them. With synergistic selection between deleterious variation, similar outcomes of predominant selfing or predominant outcrossing are expected, although highly selfing populations can maintain some genetic load (Charlesworth *et al.* 1991). Other theoretical studies have shown that linkage and identity disequilibria, *i.e.*, variation in the degree of individual heterozygosity created by variation in past ancestry of selfing and outcrossing, could result in the maintenance of genetic diversity because of selective interference between completely recessive deleterious alleles, or because of the interaction between genetic drift and selection on beneficial alleles (Lande *et al.* 1994; Roze 2015).

Our results are difficult to reconcile with these theoretical studies in that more genetic diversity was maintained under trioecy and androdioecy than expected by chance and directional selection. In principle, two mechanisms could lead to this pattern: (1) associative overdominance generated by deleterious recessive alleles at high linkage disequilibrium and in repulsion phase or, (2) heterozygote advantage generated by overdominant loci (Ohta and Kimura 1970; Palsson and Pamilo 1999). Linkage disequilibrium was low and similar under trioecy and androdioecy suggesting that the deleterious recessive alleles did not result in excess genetic diversity unless the relevant loci were very closely linked. Identity disequilibrium was on the other hand clearly important under androdioecy. Although identity disequilibrium is usually thought of as revealing homozygous correlations between different loci, it may also reveal heterozygosity correlations (Szulkin *et al.* 2010). Under androdioecy, increased identity disequilibrium might have increased fitness variance among individuals and favored multilocus heterozygotes in loci with deleterious recessive alleles or overdominant loci, which unlike with linkage disequilibrium, can be spread across the genome (Charlesworth and Charlesworth 1990; Chelo and Teotónio 2013).

If excess genetic diversity during experimental evolution resulted from overdominant loci and/or deleterious recessives in identity disequilibrium, then androdioecious populations should have maintained some of the ancestral genetic load (Charlesworth and Charlesworth 1990; Charlesworth *et al.* 1991); but they did not. It may be that the majority of unlinked deleterious recessives were initially purged because of selfing, allowing then for the expression of only a few unlinked overdominant loci that when in the homozygous state resulted in high mortality, as suggested by Nordborg *et al.* (1996). In the experimental androdioecious populations, these few overdominant loci would not necessarily generate much fitness variance, otherwise populations would have approached extinction. And although present in the trioecious populations, as we assume that all responses are from standing genetic variation, these loci would have had little opportunity to be expressed during experimental evolution. Instead, in trioecious populations, closely linked deleterious recessives, perhaps still remaining from the initial hybridization of wild isolates, could generate associative overdominance responsible for the observed excess diversity (but see Chelo and Teotónio (2013)).

Under partial selfing (androdioecy), heterozygote advantage could be also generated by recessive lethals in repulsion phase, which are effectively overdominant loci despite the potentially large physical distances between them. Distinguishing these alternatives would require mapping the relevant fitness loci in the genome, an endeavor that is notoriously difficult and beyond the scope of the present study. Recessive sub-lethals in repulsion have been described in natural populations of *C. elegans*, albeit always in strong linkage disequilibrium and at close physical distance, *e.g.*, Seidel *et al.* (2008), and are thus likely to also be present in our populations as they ultimately are derived from a cross between wild isolates (Chelo *et al.* 2013).

Monoecious populations show an overall reduction in genetic diversity that is in line with neutral expectations under exclusive selfing (Crow and Kimura 1970). It is possible, however, that some form of frequency- or density-dependent selection acted to maintain diversity in the experimental populations as it appears that there was a reduction genetic diversity during our inbreeding assay (Figure 5). In particular, populations were maintained at high densities where different genotypes could interact, while during the inbreeding assay, which was done by individual selfing, no such interactions were possible.

Our experiments involved placing populations in a novel environment in order for substantial genetic variance for fitness to be expressed (when compared to the domestication environment to which populations prior to sex determination manipulation were adapted to). We have previously shown that in the novel salt environment populations from all three reproduction systems had similar adaptive rates [Figure 4 in Theologidis *et al.* (2014)]. Yet, we also found that male competitive ability evolved under obligatory outcrossing in male-female (truly dioecious) populations (Figure 6B in Theologidis *et al.* (2014)). Sexual selection among males could thus have confounded the effects of selfing. In particular, sexual selection is an alternative mechanism to inbreeding by selfing for the purging of deleterious recessive alleles when these are preferentially expressed in the heterogametic sex (Agrawal 2001; Siller 2001). Because we have not measured genetic loads for male fitness components, whether sexual selection or reproductive mode was responsible for the evolution of standing genetic variation and purging of genetic loads is unknown and further investigation will be required. However, a recent experimental evolution study in the hermaphroditic snail *Physa acuta*, specifically designed to disentangle sexual selection from selfing in the purging of deleterious recessives, has failed to find a significant role for sexual selection (Noël *et al.* 2019).

Selfing rates under androdioecy were unstable during experimental evolution and, unless male fitness components were to evolve, exclusive selfing would be achieved in less than 100 generations (Theologidis *et al.* 2014). Although partial selfing is found in natural populations it is not particularly common (Goodwillie *et al.* 2005; Jarne and Auld 2006). It is thus reasonable to ask why partial selfing is not more common if it allows the purging of unlinked deleterious recessives while maintaining genetic diversity at a few partially dominant or overdominant loci in identity desiquilibrium, as shown here. Insights into this problem will come from a better understanding of the short-term advantages of selfing in providing reproductive assurance when outcrossing is limited (Cheptou 2012; Theologidis *et al.* 2014) or in creating the opportunity for the evolution of sexual conflicts and allocation of resources to different sexual functions (Carvalho *et al.* 2014b; Poullet *et al.* 2016), together with the long-term costs of reduced effective population sizes and recombination that may be important during sustained environmental change (Burt 2000; Igic *et al.* 2008; Goldberg *et al.* 2010; Lande and Porcher 2015).

## References

[bib1] Agrawal A. F., 2001 Sexual selection and the maintenance of sexual reproduction. Nature 411: 692–695. 10.1038/35079590 11395771

[bib2] Andersen E. C., Gerke J. P., Shapiro J. A., Crissman J. R., Ghosh R., 2012 Chromosome-scale selective sweeps shape *Caenorhabditis elegans* genomic diversity. Nat. Genet. 44: 285–290. 10.1038/ng.1050 22286215 PMC3365839

[bib3] Anderson J., Morran L., and Phillips P., 2010 Outcrossing and the Maintenance of Males within *C. elegans* Populations. J. Hered. 101: S62–S74. 10.1093/jhered/esq003 20212008 PMC2859890

[bib4] Bierne N., Tsitrone A., and David P., 2000 An inbreeding model of associative overdominance during a population bottleneck. Genetics 155: 1981–1990. 10924490 10.1093/genetics/155.4.1981 PMC1461183

[bib5] Bradic M., Costa J., and Chelo I. M., 2011, pp. 193–210 in Genotyping with Sequenom, edited by Orgogozo V., Rockman M. Humana Press, New York. 10.1007/978-1-61779-228-1_11 22065439

[bib6] Burt A., 2000 Perspective: Sex, recombination and the efficacy of selection: was Weissman right? Evolution 54: 337–351. 10937212 10.1111/j.0014-3820.2000.tb00038.x

[bib7] Carvalho S., Chelo I. M., Goy C., and Teotónio H., 2014 a The role of hermaphrodites in the experimental evolution of increased outcrossing rates in *Caenorhabditis elegans*. BMC Evol. Biol. 14: 116 10.1186/1471-2148-14-116 24891031 PMC4055231

[bib8] Carvalho S., Phillips P. C., and Teotónio H., 2014 b Hermaphrodite life history and the maintenance of partial selfing in experimental populations of *Caenorhabditis elegans*. BMC Evol. Biol. 14: 117 10.1186/1471-2148-14-117 24891140 PMC4052797

[bib9] Charlesworth B., Morgan M., and Charlesworth D., 1991 Multilocis models of inbreeding depression with synergistic selection and partial self-fertilization. Genet. Res. 57: 177–194. 10.1017/S0016672300029256

[bib10] Charlesworth D., and Charlesworth B., 1987 Inbreeding depression and its evolutionary consequences. Annu. Rev. Ecol. Evol. Syst. 18: 237–268. 10.1146/annurev.es.18.110187.001321

[bib11] Charlesworth D., and Charlesworth B., 1990 Inbreeding depression with heterozygote advantage and its effect on selection for modifiers changing the outcrossing rate. Evolution 44: 870–888. 10.1111/j.1558-5646.1990.tb03811.x 28569012

[bib12] Charlesworth D., Morgan M. T., and Charlesworth B., 1990 Inbreeding depression, genetic load, and the evolution of outcrossing rates in a multilocus system with no linkage. Evolution 44: 1469–1489. 10.1111/j.1558-5646.1990.tb03839.x 28564321

[bib13] Chelo I. M., Carvalho S., Manoel D., Proulx S., and Teotónio H., 2013 The genetic basis and experimental evolution of inbreeding depression in Caenorhabiditis elegans. Heredity 112: 248–254. 10.1038/hdy.2013.100 24129606 PMC3931175

[bib14] Chelo I. M., and Teotónio H., 2013 The opportunity for balancing selection in experimental populations of *Caenorhabditis elegans*. Evolution 67: 142–156. 10.1111/j.1558-5646.2012.01744.x 23289568

[bib15] Cheptou P. O., 2012 Clarifying BakerÂ’s Law. Ann. Bot. 109: 633–641. 10.1093/aob/mcr127 21685434 PMC3278284

[bib16] Cox D. R., 1972 Regression models and life tables. J. R. Stat. Soc. B 34: 187–220.

[bib17] Crow J. F., and Kimura M., 1970 An Introduction to Population Genetics Theory, Harper & Row, Publishers, New York.

[bib18] Cutter A. D., 2006 Nucleotide polymorphism and linkage disequilibrium in wild populations of the partial selfer *Caenorhabditis elegans*. Genetics 172: 171–184. 10.1534/genetics.105.048207 16272415 PMC1456145

[bib19] David P., Pujol B., Viard F., Castella V., and Goudet J., 2007 Reliable selfing rate estimates from imperfect population genetic data. Mol. Ecol. 16: 2474–2487. 10.1111/j.1365-294X.2007.03330.x 17561907

[bib20] Dolgin E. S., Charlesworth B., Baird S. E., and Cutter A. D., 2007 Inbreeding and outbreeding depression in Caenorhabditis nematodes. Evolution 61: 1339–1352. 10.1111/j.1558-5646.2007.00118.x 17542844

[bib21] Estes S., Phillips P. C., Denver D. R., Thomas W. K., and Lynch M., 2004 Mutation accumulation in populations of varying size: the distribution of mutational effects for fitness correlates in *Caenorhabditis elegans*. Genetics 166: 1269–1279. 10.1534/genetics.166.3.1269 15082546 PMC1470770

[bib22] Gaertner B. E., Parmenter M. D., Rockman M. V., Kruglyak L., and Phillips P. C., 2012 More than the sum of its parts: a complex epistatic network underlies natural variation in thermal preference behavior in *Caenorhabditis elegans*. Genetics 192: 1533–1542. 10.1534/genetics.112.142877 23086219 PMC3512158

[bib23] Goldberg E. E., Kohn J. R., Lande R., Robertson K. A., Smith S. A., 2010 Species selection maintains self-incompatibility. Science 330: 493–495. 10.1126/science.1194513 20966249

[bib24] Goodwillie C., Kalisz S., and Eckert C. G., 2005 The evolutionary enigma of mixed mating systems in plants: occurrence, theoreticalexplanations, and empirical evidence. Annu. Rev. Ecol. Evol. Syst. 36: 47–79. 10.1146/annurev.ecolsys.36.091704.175539

[bib25] Guzella T. S., Dey S., Chelo I. M., Pino-Querido A., Pereira V. F., 2018 Slower environmental change hinders adaptation from standing genetic variation. PLoS Genet. 14: e1007731 10.1371/journal.pgen.1007731 30383789 PMC6233921

[bib26] Haldane J. B. S., 1949 The association of characters as a result of inbreeding and linkage. Ann. Eugen. 15: 15–23. 10.1111/j.1469-1809.1949.tb02418.x 15403124

[bib27] Igic B., Lande R., and Kohn J. R., 2008 Loss of Self-Incompatibility and Its Evolutionary Consequences. Int. J. Plant Sci. 169: 93–104. 10.1086/523362

[bib28] Jarne P., and Auld J. R., 2006 Animals mix it up too: the distribution of self-fertilization among hermaphroditic animals. Evolution 60: 1816–1824. 10.1111/j.0014-3820.2006.tb00525.x 17089966

[bib29] Johnston M. O., Porcher E., Cheptou P. O., Eckert C. G., Elle E., 2009 Correlations among fertility components can maintain mixed mating in plants. Am. Nat. 173: 1–11. 10.1086/593705 19055444

[bib30] Kaplan E. L., and Meier P., 1958 Nonparametric estimation from incomplete observations. J. Am. Stat. Assoc. 53: 457–481. 10.1080/01621459.1958.10501452

[bib31] Kelly J. K., 2007 Mutation-selection balance in mixed-mating populations. J. Theor. Biol. 246: 355–365. 10.1016/j.jtbi.2006.12.030 17291538 PMC2040123

[bib32] Kimura M., 1983 The Neutral Theory of Molecular Evolution, Cambridge University Press, Cambridge 10.1017/CBO9780511623486

[bib33] Lande R., and Porcher E., 2015 Maintenance of Quantitative Genetic Variance Under Partial Self-Fertilization, with Implications for Evolution of Selfing. Genetics 200: 891–906. 10.1534/genetics.115.176693 25969460 PMC4512550

[bib34] Lande R., and Schemske D. W., 1985 The evolution of self-fertilization and inbreeding depression in plants. I. Genetic models. Evolution 39: 24–40. 10.1111/j.1558-5646.1985.tb04077.x 28563655

[bib35] Lande R., Schemske D. W., and Schultz S. T., 1994 High inbreeding depression, selective interference among loci, and the threshold selfing rate for purging recessive lethal mutations. Evolution 48: 965–978. 10.1111/j.1558-5646.1994.tb05286.x 28564486

[bib36] Lenth R. V., 2015 lsmeans: Least-Squares Means. R package version 2.20–23. http://CRAN.R-project.org/package=lsmeans.

[bib37] Lynch M., and Walsh B., 1998 Genetics and Analysis of Quantitative Traits, Sinauer Associates, Sunderland, MA.

[bib38] Masri L., Schulte R. D., Timmermeyer N., Thanisch S., Crummenerl L. L., 2013 Sex differences in host defence interfere with parasite-mediated selection for outcrossing during host-parasite coevolution. Ecol. Lett. 16: 461–468. 10.1111/ele.12068 23301667 PMC3655609

[bib39] Matuszewski S., Hermisson J., and Kopp M., 2015 Catch Me if You Can: Adaptation from Standing Genetic Variation to a Moving Phenotypic Optimum. Genetics 200: 1255–1274. 10.1534/genetics.115.178574 26038348 PMC4574244

[bib40] Noble L. M., Chelo I., Guzella T., Afonso B., Riccardi D. D., 2017 Polygenicity and Epistasis Underlie Fitness-Proximal Traits in the Caenorhabditis elegans Multiparental Experimental Evolution (CeMEE) Panel. Genetics 207: 1663–1685. 10.1534/genetics.117.300406 29066469 PMC5714472

[bib41] Nordborg M., Charlesworth B., and Charlesworth D., 1996 Increased levels of polymorphim surrounding selectively maintained sites in highly selfing species. Proc. Biol. Sci. 263: 1033–1039. 10.1098/rspb.1996.0152

[bib42] Noël E., Fruitet E., Lelaurin D., Bonel N., Ségard A., 2019 Sexual selection and inbreeding: Two efficient ways to limit the accumulation of deleterious mutations. Evolution Letters 3: 80–92. 10.1002/evl3.93 30788144 PMC6369961

[bib43] Ohta T., and Kimura M., 1970 Development of associative overdominance through linkage disequilibrium in finite populations. Genet. Res. 16: 165–177. 10.1017/S0016672300002391 5516427

[bib44] Palsson S., and Pamilo P., 1999 The effects of deleterious mutations on linked, neutral variation in small populations. Genetics 153: 475–483. 10471727 10.1093/genetics/153.1.475 PMC1460752

[bib45] Pinheiro J., Bates D., DebRoy S., Sarkar D., and R Core Team, 2018 *nlme: Linear and Nonlinear Mixed Effects Models*. R package version 3.1–137.

[bib46] Poullet N., Vielle A., Gimond C., Carvalho S., Teotónio H., 2016 Complex heterochrony underlies the evolution of *Caenorhabditis elegans* hermaphrodite sex allocation. Evolution 70: 2357–2369. 10.1111/evo.13032 27501095

[bib47] Rockman M., and Kruglyak L., 2009 Recombinational Landscape and Population Genomics of *Caenorhabditis elegans*. PLoS Genet. 5: e1000419 10.1371/journal.pgen.1000419 19283065 PMC2652117

[bib48] Rockman M. V., Skrovanek S. S., and Kruglyak L., 2010 Selection at linked sites shapes heritable phenotypic variation in *C. elegans*. Science 330: 372–376. 10.1126/science.1194208 20947766 PMC3138179

[bib49] Roze D., 2015 Effects of Interference Between Selected Loci on the Mutation Load, Inbreeding Depression, and Heterosis. Genetics 201: 745–757. 10.1534/genetics.115.178533 26269503 PMC4596681

[bib50] Roze D., 2016 Background selection in partially selfing populations. Genetics 203: 937–957. 10.1534/genetics.116.187955 27075726 PMC4896204

[bib51] Schultz S. T., and Lynch M., 1997 Mutation and extinction: the role of variable mutational effects, synergistic epistasis, beneficial mutations, and degree of outcrossing. Evolution 51: 1363–1371. 10.1111/j.1558-5646.1997.tb01459.x 28568635

[bib52] Seidel H. S., Rockman M. V., and Kruglyak L., 2008 Widespread genetic incompatibility in *C. elegans* maintained by balancing selection. Science 319: 589–594. 10.1126/science.1151107 18187622 PMC2421010

[bib53] Siller S., 2001 Sexual selection and the maintenance of sex. Nature 411: 689–692. 10.1038/35079578 11395770

[bib54] Skaug H., Fournier D., Nielsen A., Magnusson A., and Bolker B., 2013 Generalized lineag mixed models using AD Model Builder. R package version 0.7.5.

[bib55] Stewart A. D., and Phillips P. C., 2002 Selection and maintenance of androdioecy in *Caenorhabditis elegans*. Genetics 160: 975–982. 11901115 10.1093/genetics/160.3.975 PMC1462032

[bib56] Stiernagle T., 1999 Maintenance of C. elegans, Oxford University Press, Oxford.

[bib57] Stoffel M. A., Esser M., Kardos M., Humble E., Nichols H., 2016 inbreedr: An r package for the analysis of inbreeding based on genetic markers. Methods Ecol. Evol. 7: 1331–1339. 10.1111/2041-210X.12588

[bib58] Sved J. A., 1971 Linkage disequilibrium and homozygosity of chromosome segments in finite populations. Theor. Popul. Biol. 2: 125–141. 10.1016/0040-5809(71)90011-6 5170716

[bib59] Szulkin M., Bierne N., and David P., 2010 Heterozygosity-fitness correlations: a time for reappraisal. Evolution 64: 1202–1217. 10.1111/j.1558-5646.2010.00966.x 20148954

[bib60] Teotónio H., Carvalho S., Manoel D., Roque M., and Chelo I. M., 2012 Evolution of outcrossing in experimental populations of *Caenorhabditis elegans*. PLoS One 7: e35811 10.1371/journal.pone.0035811 22540006 PMC3335146

[bib61] Teotónio H., Estes S., Phillips P., and Baer C. F., 2017 Experimental evolution with Caenorhabditis nematodes. Genetics 206: 691–716. 10.1534/genetics.115.186288 28592504 PMC5499180

[bib62] Teotónio H., Manoel D., and Phillips P., 2006 Genetic variation for outcrossing among *Caenorhabditis elegans* isolates. Evolution 60: 1300–1305. 10.1111/j.0014-3820.2006.tb01207.x 16892979

[bib63] Theologidis I., Chelo I. M., Goy C., and Teotónio H., 2014 Reproductive assurance drives transitions to self-fertilization in experimental *Caenorhabditis elegans*. BMC Biol. 12: 93 10.1186/s12915-014-0093-1 25369737 PMC4234830

[bib64] Therneau T., 2015 coxme: mixed effects cox models. R package version 2–2.5.

[bib65] Vassilieva L. L., Hook A. M., and Lynch M., 2000 The fitness effects of spontaneous mutations in *Caenorhabditis elegans*. Evolution 54: 1234–1246. 10.1111/j.0014-3820.2000.tb00557.x 11005291

[bib66] Weir B., and Cockerham C. C., 1973 Mixed self and random mating at two loci. Genet. Res. 21: 247–262. 10.1017/S0016672300013446 4731639

[bib67] Weir B. S., 1996 Genetic data analysis II, Sinauer Associates, Sunderland, MA.

[bib68] Ziehe M., and Roberds J. H., 1989 Inbreeding depression due to overdominance in partially self-fertilizing plant populations. Genetics 121: 861–868. 17246494 10.1093/genetics/121.4.861 PMC1203670

